# Characterisation of tumour blood flow using a 'tissue-isolated' preparation.

**DOI:** 10.1038/bjc.1994.445

**Published:** 1994-12

**Authors:** G. M. Tozer, K. M. Shaffi, V. E. Prise, V. J. Cunningham

**Affiliations:** CRC Gray Laboratory, Mount Vernon Hospital, Northwood, Middlesex, UK.

## Abstract

Tumour blood flow was characterised in a 'tissue-isolated' rat tumour model, in which the vascular supply is derived from a single artery and vein. Tumours were perfused in situ and blood flow was calculated from simultaneous measurement of (1) venous outflow from the tumour and (2) uptake into the tumour of radiolabelled iodo-antipyrine (IAP). Comparison of results from the two measurements enabled assessment of the amount of blood 'shunted' through the tumours with minimal exchange between blood and tissue. Kinetics of IAP uptake were also used to determine the apparent volume of distribution (VDapp) for the tracer and the equilibrium tissue-blood partition coefficient (lambda). lambda was also measured by in vitro techniques and checks were made for binding and metabolism of IAP using high-pressure liquid chromatography. VDapp and lambda were used to calculate the perfused fraction (alpha) of the tumours. Tumour blood flow, as measured by IAP (TBFIAP), was 94.8 +/- 4.4% of the blood flow as measured by venous outflow, indicating only a small amount of non-exchanging flow. This level of shunting is lower than some previous estimates in which the percentage tumour entrapment of microspheres was used. The unperfused fraction ranged from 0 to 20% of the tumour volume in the majority of tumours. This could be due to tumour necrosis and/or acutely ischaemic tumour regions. For practical purposes, measurement of the total venous outflow of tumours is a reasonable measure of exchangeable tumour blood flow in this system and allows for on-line measurements. Tracer methods can be used to obtain additional information on the distribution of blood flow within tumours.


Br. J. Cancer (1994), 70, 1040-1046 © Macmillan Press Ltd., 1994

### Characterisation of tumour blood flow using a 'tissue-isolated' preparation

G.M. Tozer[1], K.M. Shaffi[1], V.E. Prise[1] & V.J. Cunningham[2]

[1]CRC Gray Laboratory, PO Box 100, Mount Vernon Hospital, Northwood, Middlesex HA6 2JR, UK; [2]MRC Cyclotron Unit, Hammersmith Hospital, DuCane Road, London W12 0HS, UK.

**Summary** Tumour blood flow was characterised in a 'tissue-isolated' rat tumour model, in which the vascular supply is derived from a single artery and vein. Tumours were perfused *in situ* and blood flow was calculated from simultaneous measurement of (1) venous outflow from the tumour and (2) uptake into the tumour of radiolabelled iodo-antipyrine (IAP). Comparison of results from the two measurements enabled assessment of the amount of blood 'shunted' through the tumours with minimal exchange between blood and tissue. Kinetics of IAP uptake were also used to determine the apparent volume of distribution ($VD_{app}$) for the tracer and the equilibrium tissue–blood partition coefficient ($\lambda$). $\lambda$ was also measured by *in vitro* techniques and checks were made for binding and metabolism of IAP using high-pressure liquid chromatography. $VD_{app}$ and $\lambda$ were used to calculate the perfused fraction ($\alpha$) of the tumours. Tumour blood flow, as measured by IAP ($TBF_{IAP}$), was $94.8 \pm 4.4\%$ of the blood flow as measured by venous outflow, indicating only a small amount of non-exchanging flow. This level of shunting is lower than some previous estimates in which the percentage tumour entrapment of microspheres was used. The unperfused fraction ranged from 0 to 20% of the tumour volume in the majority of tumours. This could be due to tumour necrosis and/or acutely ischaemic tumour regions. For practical purposes, measurement of the total venous outflow of tumours is a reasonable measure of exchangeable tumour blood flow in this system and allows for on-line measurements. Tracer methods can be used to obtain additional information on the distribution of blood flow within tumours.

Tumours become vascularised under the influence of tumour-derived angiogenic factors (Folkman, 1985). After vessel formation, tumour growth has a large influence on the subsequent vascular pattern. Rubin and Casarett (1966) described two types of tumour vascularisation, a central type with vessels branching outwards from the tumour centre and a peripheral type where the largest vessels are apparent at the tumour periphery and necrosis tends to develop centrally. Falk (1980) proposed that central vascularisation is the more basic form from which the peripheral form may develop as a result of the 'stresses and strains' of tumour growth. Examples of the effects of such stresses and strains include the characteristic tortuosity of tumour blood vessels, occlusion of vessels and dilatation of vessels upstream from occluded sites, all of which are likely to be induced by pressure from division of surrounding tumour parenchymal cells (Warren, 1979; Falk, 1980; Shubik, 1982). Capillary lengthening is another commonly observed feature of tumours and is probably caused by stretching forces of the growing tumour as well as incorporation of tumour parenchymal cells into the vessel wall (Endrich *et al.*, 1982; Dewhirst *et al.*, 1989). Such vessels are thus often devoid of true endothelial cell linings which could, in turn, lead to platelet and leucocyte adherence.

In view of these anatomical characteristics, it is not surprising that both spatial and temporal heterogeneity of tumour blood flow have been reported (Chaplin *et al.*, 1987; Tozer *et al.*, 1990). Regions of very low blood flow may result from large intercapillary spaces and/or occluded blood vessels and these regions would be unfavourable for the uptake of chemotherapeutic drugs into tumours. It has also been proposed that conditions in the growing tumour described above could give rise to the development of arteriovenous anastomoses (Tveit *et al.*, 1987; Vaupel *et al.*, 1989) resulting in a high proportion of 'shunted' blood from which there is little exchange with the surrounding tumour cells. Again, this would be a barrier to effective chemotherapy.

We have used a 'tissue-isolated' preparation, in which the tumour microcirculation is derived from a single artery and vein, in order to investigate the perfused fraction and level of shunted blood flow in the P22 rat carcinosarcoma perfused *in situ*. Venous outflow from the tumour, which measures total tumour blood flow, was compared with blood flow measured by the uptake of radiolabelled iodo-antipyrine (IAP), for assessment of shunting. External gamma counting of tumour levels of IAP at long times (up to 2 h) after administration of IAP allowed the measurement of the tissue–blood partition coefficient of the tracer ($\lambda$). The apparent volume of distribution for IAP ($VD_{app}$) was calculated from the kinetics of its uptake at a short time (400 s) after administration. Comparison of $\lambda$ and $VD_{app}$ allowed calculation of a perfused fraction for the tumour ($\alpha$). $\lambda$ was also measured separately, under *in vitro* conditions, for comparison. High-performance liquid chromatography (HPLC) measurements of IAP levels in tumour and blood were made to check for any metabolism of IAP which could affect the interpretation of the *in vivo* data.

### Materials and methods

### Tumours

A transplanted rat carcinosarcoma, designated P22, was used for these experiments. Its origin and maintenance in BD9 rats have been described previously (Tozer & Shaffi, 1993). Early-generation tumours (third to tenth generation away from the primary tumour), growing subcutaneously in the flanks of male BD9 rats, were excised and used as donor material for the propagation of tissue-isolated tumours in recipient male BD9 rats. This type of preparation was first described by Gullino and Grantham (1961*a*) and in the inguinal site by Grantham *et al.* (1973). We have previously used tissue-isolated tumours for *ex vivo* perfusions with physiological buffer, in order to investigate the relationship between flow and perfusion pressure (Sensky *et al.*, 1993). We now describe a system for *in vivo* perfusion with blood. Recipient rats were anaesthetised by intraperitoneal injection of fentanyl citrate ($0.315\ mg\ kg^{-1}$) and fluanisone ($10\ mg\ kg^{-1}$) (Hypnorm, Crown Chemical and midazolam ($5\ mg\ kg^{-1}$) (Hypnovel, Roche). A small incision was made in the skin over the right hind leg and a small section ($\approx 0.2$ g) of the right inguinal fat pad was surgically isolated together with the proximal portion of the right superior epigastric artery and vein which branch from the right femoral artery and vein respectively.

Donor tumour was implanted into the isolated fat as a slurry prepared by mincing the donor tumour with scissors

Correspondence: G. Tozer.
Received 26 May 1994; and in revised form 25 July.

followed by aspiration through hypodermic needles of decreasing sizes. Moulded silicon chambers made from Silastic MDX4-4210 medical grade elastomer (Dow Corning) were used to enclose the fat and growing tumour in order to prevent ingrowth of new blood vessels from surrounding fat, skin or muscle. The chambers were constructed so as not to constrict the vascular pedicle and to allow drainage of fluid. Chambers were loosely sutured to subcutaneous fat to prevent them twisting in the inguinal cleft and thus constricting the vascular pedicle. Wounds were stitched and teeth were clipped to prevent the animals from interfering with their stitches. Lost fluid from evaporation was replaced by subcutaneous injection of several millilitres of dextrose/saline solution and rats were placed on a heated blanket until partial recovery from the effects of anaesthesia. They were subsequently housed in separate cages with free access to both soft and regular diet and water. Three to four days after the operation rats were reanaesthetised lightly with halothane and the teeth reclipped.

*Surgical preparation*

Tumour growth was assessed qualitatively by palpation and tumours grew to fill the chambers within 2–3 weeks of implantation. Tumours could grow to a maximum size of approximately 0.8 g within the chambers. When larger tumours were required animals were reanaesthetised with halothane and nitrous oxide in oxygen, a skin incision was made and part of the chamber was cut away to allow further tumour growth. Wounds were stitched and teeth were clipped. Growth could proceed in this way for several days without any ingrowth of new blood vessels.

Animals were prepared for experimentation when tumours reached 0.5–1.5 g in weight. Animals were reanaesthetised with Hypnorm and midazolam and the skin overlying the tumour reopened. The surgical procedure is shown in Figure 1. The saphenous artery was catheterised with a polyethylene catheter (internal diameter 0.28 mm, Portex) such that the catheter tip passed along the femoral artery until it was just distal to the tumour (superior epigastric) artery. The femoral vein was catheterised using wide-bore silicon tubing with a narrower polyethylene tip (internal diameter 0.58 mm, Portex) for insertion into the vein. The catheter was positioned such that its tip was just distal to the tumour (superior epigastric) vein. A piece of suture thread was placed under the femoral vein proximal to the tumour vein. All other vessels near the catheterisation site, including the saphenous vein and muscular artery and vein, were ligated or cauterised.

**Figure 1** Surgical preparation of the vasculature in the right inguinal cleft of the BD9 rat for *in situ* isolation of the tumour blood supply. Small blood vessels not shown in the diagram are occluded by cauterisation. Not to scale.

The catheterisation procedure permitted the administration of IAP directly to the tumour without its entry into the systemic circulation and without interference with the blood supply to the tumour from the femoral artery. It also enabled collection of venous outflow from the tumour without disrupting its arterial supply. The tumour was kept warm and moist throughout the surgical procedure. A tail artery and two tail veins were also catheterised using polyethylene tubing (internal diameter 0.58 mm, Portex) and the wounds strapped. Mean arterial blood pressure (MABP) was monitored continuously via a physiological pressure transducer (Gould Instruments Systems) connected to the tail artery catheter. The preparation was left undisturbed for approximately half an hour before further experimentation. MABP, for the whole group of animals, was $87.0 \pm 0.3$ mmHg immediately after catheterisation and did not vary significantly throughout the course of the experiment.

*Isolation of tumour blood supply and measurement of venous outflow*

After the rest period, rats were heparinised by intravenous bolus injection via the tail vein catheter, (0.1 ml heparin, 1,000 units $ml^{-1}$, CP Pharmaceuticals). Venous drainage from the tumour was diverted from the femoral vein to the venous catheter by tying off the suture around the femoral vein. Venous blood was collected every 1–2 min into preweighed vials with the aid of a fraction collector. Continuous monitoring of venous outflow in ml $min^{-1}$ was obtained by weighing each blood sample and using a value of 1.05 for the density of whole blood. Tumour weight, measured at the end of the experiment, was used to calculate venous outflow in ml $g^{-1}$ $min^{-1}$.

Blood volume of the rat was maintained constant by infusion of donor blood*, via a peristaltic pump, into a catheterised tail vein. The rate of infusion was continually adjusted to equal the venous outflow measured in the previous 1–2 min time interval. Despite the delay involved here, there was never more than a 5% discrepancy between venous outflow and rate of blood replacement over the time course of each experiment. This difference had no effect on systemic blood pressure. The reservoir of donor blood was stirred continuously. Top-up doses of anaesthetic were administered intraperitoneally at regular intervals and rats were kept warm using an angle-poise lamp and a thermostatically controlled heating blanket connected to a rectal thermocouple. Surface tumour temperature was monitored using a second thermocouple and maintained between 34.0 and 36.5°C throughout the experimental period using the lamp.

*Measurement of tumour blood flow using IAP ($TBF_{IAP}$)*

$TBF_{IAP}$ was measured using the uptake of radiolabelled IAP into the tumour in one of two ways. In the first method, tumours were shielded from the rest of the rat by placing them on a specially constructed lead platform which enabled a gamma scintillation counter (diameter 1.2 cm), with lead

*Blood was obtained from donor animals 1–5 days before the experiment. Donor animals were anaesthetised with Hypnorm and midazolam for catheterisation of the right carotid artery. Between 10 and 20 ml of free-flowing arterial blood was collected from each animal under terminal anaesthesia into citrate–phosphate–dextrose (CPD) solution [0.14 ml of CPD (Sigma Chemical Co., Poole, Dorset, UK) to 1 ml of whole blood]. Blood samples were refrigerated until required and then recalcified using 0.02 ml of calcium gluconate (10% solution) per ml of whole blood and heparinised using 0.005 ml of heparin (1,000 units $ml^{-1}$) per ml of whole blood. Blood samples were then spun down and part of the supernatant removed in order to adjust the haematocrit to that of the recipient rat. Red cells were resuspended and the samples warmed to 37°C in a water bath ready for infusion into the recipient rat.

collimating sleeve, to be placed over the tumour without stretching or constriction of the tumour vascular pedicle. $^{125}$I-labelled IAP ($^{125}$I-IAP, Amersham), at a concentration of 0.074–0.37 MBq (2–10 $\mu$Ci) per ml of saline, was given as a constant infusion to the tumour via the arterial catheter at approximately 5% (never more than 7%) of the rate of the total venous outflow. Tumour levels of IAP were measured continuously via the gamma counter and recorded on a Macintosh IIci computer using electronic interfaces constructed 'in-house'. Venous levels of IAP (c.p.m. g$^{-1}$) draining the tumour were measured from timed venous samples counted in a well-type scintillation counter (LKB). At the end of the experiment (1–2 h after the start of IAP infusion) rats were killed using bolus intravenous injection of Euthatal (RMB Animal Health) and the tumour rapidly excised, weighed and counted in the well counter. These counts were used to determine a factor which was used to convert all the data from the external counter into equivalent well counter counts. Venous outflow (ml min$^{-1}$) was monitored continuously throughout the experiment.

In the second method for measuring TBF$_{IAP}$, IAP was infused systemically over a short time period and tumour levels of IAP were measured after excision, by scintillation counting. This method has been published previously for subcutaneous P22 tumours (Tozer & Shaffi, 1993). Briefly, the tail artery catheter was disconnected from the pressure transducer and connected to a fraction collector loaded with preweighed vials and 0.37 MBq (10 $\mu$Ci) of $^{125}$I-IAP or $^{14}$C-labelled IAP ($^{14}$C-IAP) in 0.8 ml of saline was infused into the second catheterised tail vein over a period of 30 s. During the 30 s period free-flowing arterial blood from the tail artery catheter was collected into preweighed vials at 1 s intervals. At the end of 30 s the tumour vascular pedicle was ligated close to the tumour and the tumour excised. Venous outflow from the tumour was monitored continuously throughout the experiment. The tumour and blood samples were weighed and counted using the well counter for samples containing $^{125}$I and liquid scintillation for samples containing $^{14}$C as described by Tozer and Shaffi (1993). Measurement of venous outflow from the tumour was continued for approximately 10 min after excision of the tumour in order to determine the level of residual flow in the vascular pedicle. The rat was then killed using a bolus intravenous injection of Euthatal.

*Calculation of TBF$_{IAP}$*

Calculations of TBF$_{IAP}$ from data obtained using the two methods described above were based on the Kety (1960) model:

$$C_{tiss}(t) = k_1 C_a(t) \otimes e^{-k_2 t} \quad (1)$$

where $C_{tiss}(t)$ is concentration of label in the tumour as a function of time, $t$, in c.p.m. g$^{-1}$, $k_1$ is TBF$_{IAP}$ per g of whole tumour in ml g$^{-1}$ min$^{-1}$, $C_a(t)$ is the concentration of label in the arterial blood entering the tumour as a function of $t$, in c.p.m. ml$^{-1}$, $\otimes$ denotes the convolution integral, and $k_2 = k_1/\alpha\lambda$ where $\alpha$ is the fraction of tumour effectively perfused and $\lambda$ is the equilibrium partition coefficient of the tracer between tissue and blood ($\alpha\lambda$ is equivalent to VD$_{app}$, the *apparent* volume of distribution of the label in the tissue relative to the blood).

In the first method $C_{tiss}(t)$ was measured continuously over the experimental period, and the concentration of label in blood at the arterial infusion site [$C_i(t)$, c.p.m. ml$^{-1}$] was calculated from the infusion rate of IAP into the artery, the concentration of IAP in the infusate and the rate of venous outflow of blood from the tumour, also measured throughout the experiment. The relationship between $C_i(t)$ and $C_a(t)$ involves a short delay before the tracer reaches the tumour and a dispersion or smearing of the tracer as it mixes with the arterial blood. Such effects are important at early times in blood flow measurements (Lammertsma *et al.*, 1990). Delay was estimated by comparing the time of start of infusion with the initial rise in tumour activity and the time scales of the blood and tissue measurements were adjusted accordingly. An exponential model of dispersion in blood sampling has been described by Iida *et al.* (1986). In the present model we have applied such a correction to the delivery of tracer to the tumour, such that:

$$C_a(t) = C_i(t) \otimes k_d e^{-k_d t} \quad (2)$$

where $k_d$ is a dispersion constant.

Combination of equations (1) and (2) gives the final model equation for the first method as:

$$C_{tiss}(t) = (k_1 k_d)/(k_d - k_2)\ C_i(t) \otimes \{e^{-k_2 t} - e^{k_d t}\} \quad (3)$$

Least mean squares estimates of $k_1$, $k_2$ and $k_d$ were obtained from a non-linear regression of $C_{tiss}(t)$ on $C_i(t)$ using equation (3) on data measured over the first 400 s of infusion of IAP as described above. The residual sum of squares was minimised using a simplex algorithm (Nelder & Mead, 1965). VD$_{app}$ was calculated from the estimates of $k_1$ and $k_2$ (see above). $\lambda$ was measured, where possible, from the ratio of tissue to blood levels of IAP at the end of the experiment (1–2 h after start of infusion). The perfused fraction, $\alpha$, is equivalent to the ratio VD$_{app}/\lambda$.

In the second method, calculation of TBF$_{IAP}$ was again based on equation (1). Here the concentration of IAP was measured in excised tumours following a short intravenous systemic infusion of the tracer (30 s), and the arterial concentration of tracer was measured from continuous tail artery sampling [$C_m(t)$]. Here the delay and dispersion effects occur in the arterial sampling catheter, such that:

$$C_m(t) = C_a(t) \otimes k_d e^{-k_d t} \quad (4)$$

In this method, $C_{tiss}(t)$ is measured at only one time point, i.e. in the tumour after excision. Hence only one parameter, $k_1$ = TBF$_{IAP}$, can be estimated from the data. VD$_{app}$ was assumed to be 0.74 (see Results section). Over such a short time period the method is relatively insensitive to small changes in VD$_{app}$ (Lammertsma *et al.*, 1992). Values for delay and dispersion in the plastic catheters were obtained from measurements made *in vitro* (G.M. Tozer & V.J. Cunningham, unpublished data). $C_m(t)$ was then deconvolved to give $C_a(t)$ (equation 4). The expected value of $C_{tiss}(t)$ at 30 s was then calculated over a range of blood flow rates, using equation (1), to give a look-up table for each individual tumour, from which the corresponding TBF$_{IAP}$ was obtained (Tozer & Shaffi, 1993).

In vitro *measurement of tissue–blood partition coefficient ($\lambda$) for $^{125}$I-IAP*

Plasma samples were obtained by centrifugation of whole blood collected, by carotid artery catheterisation, from heparinised BD9 rats under terminal anaesthesia. Approximately 0.111 MBq (3 $\mu$Ci) of $^{125}$I-IAP was added to each ml of plasma.

Five tissue-isolated, freshly excised tumours were used. Each tumour was cut into two pieces symmetrically about the vascular pedicle. The two pieces were weighed and incubated in $^{125}$I-labelled plasma for 2–3 h at 37°C to allow diffusion of IAP into the tissue samples. At the end of the equilibration period one tumour piece from each tumour as well as the appropriate plasma sample was counted using the well counter. The second piece from each tumour was frozen in isopentane at −40 to −50°C for cryostat sectioning. Sections 20 $\mu$m thick were obtained from the centre of each tumour piece and used to produce autoradiograms of IAP distribution on X-ray film as described by Tozer and Shaffi (1993).

Autoradiograms were used solely to check for uniformity of IAP distribution throughout the tumour pieces. This was apparent for all five tumours. $\lambda$ for $^{125}$I-IAP was calculated from tissue counts per g divided by plasma counts per g obtained from the remaining tumour pieces.

*Determination of IAP metabolism*

A rat bearing a tissue-isolated tumour was catheterised as described above for isolation of tumour blood supply. Venous outflow was collected during continuous infusion of $^{125}$I-IAP into the tumour artery as described above. At intervals throughout the infusion, 100 µl whole blood from the venous outflow was frozen in isopentane at $-40$ to $-50$°C. At the end of the experiment the excised tumour was also frozen. Sample were stored at $-70$°C before further analysis.

A 1 ml aliquot of methanol was added to each of the thawed blood samples, which were mixed throroughly and centrifuged. Supernatants were removed and pellets were re-extracted. The supernatants were pooled, dried under nitrogen and redissolved in 200 µl of water. Tumour samples were weighed and homogenised in four volumes of water. A 3 ml aliquot of methanol was added to a 500 µl aliquot of tumour/water mixture and extracted as described for the blood samples.

A gradient separation of the blood and tumour samples was carried out by high-performance liquid chromatography (HPLC) using a Hichrom RPB column and water (A) and 75% acetonitrile (B) as the two eluents. The linear gradient ran from 30 to 70% B in 5 min and the flow rate was 1.5 ml min$^{-1}$. Iodo-antipyrine eluted at 5–6 min. Fractions of the eluate were collected at 1 min intervals and counted in the well counter.

*Statistics*

Errors associated with mean values are one standard error of the mean. Straight lines were fitted by regression analysis with correlation coefficients ($R$) shown on the graphs. $F$-tests were used to determine whether linear fits were statistically better fits for the data than the overall mean. $P$-values of less than 0.05 were considered to be significant. Standard errors of combinations of parameters were calculated using the rules summarised by Wilkinson (1961).

**Figure 2** Two examples (tumour A and tumour B) of the uptake of $^{125}$I-IAP into the P22 tumour growing as a tissue-isolated preparation. Open symbols (○) represent tumour levels of $^{125}$I-IAP and closed symbols (■) represent venous blood levels. $\lambda$ was calculated to be $0.708 \pm 0.002$ for tumour A and $0.712 \pm 0.005$ for tumour B.

### Results

Figure 2 shows two examples (tumour A and tumour B) of the time course for $^{125}$I-IAP uptake into tumour tissue during a constant infusion of tracer into the arterial blood directly supplying the tumour. The corresponding activities in venous blood draining the two tumours are also shown. The similar shapes of the venous and tissue curves are consistent with the Kety model (see above), which assumes rapid equilibration of tracer between blood and tissue. The data show a rapid initial uptake phase followed by a plateau phase, which is consistent with the expected behaviour of a readily diffusible tracer. The equilibrium partition coefficient of IAP between tissue and blood ($\lambda$) was calculated from equilibrium levels of IAP during the plateau phase (equal to tumour counts ÷ venous blood counts) and was found to be very similar for the two tumours (Figure 2).

$TBF_{IAP}$ and $VD_{app}$, for each tumour, were determined from the first 400 s of the calculated arterial blood levels and the measured tumour levels of IAP (see Materials and methods). The first 400 s of the tumour data shown in Figure 2 are replotted in Figure 3 together with the calculated arterial blood levels at the infusion point. These data represent $C_{tiss}(t)$ and $C_i(t)$ respectively. Corresponding best fits to the tumour data are shown by the solid lines. Comparison of these data for the two tumours (A and B) shows that, despite similar values for $\lambda$, the initial uptake kinetics for the two tumours were rather different. Tumour levels of IAP for tumour A plateaued quickly and were tending to a relatively high fraction of the arterial blood IAP levels. This is reflected by relatively high values for $TBF_{IAP}$ and $VD_{app}$ respectively, compared with those for tumour B, as shown in Figure 3. $VD_{app}$ for tumour A is very similar to its $\lambda$, leading to a perfused fraction, $\alpha$, equal to 1.05 ($\alpha = VD_{app}/\lambda$). A lower $VD_{app}$ for tumour B leads to a lower perfused fraction for this tumour ($\alpha = 0.81$). Identical calculations were made for the other 11 tumours in this group.

$\lambda$, for 9 out of 13 animals, was $0.74 \pm 0.04$, which is not significantly different from the value of $0.71 \pm 0.01$ ($n = 5$) calculated from *in vitro* exposure of tumour tissue and blood to $^{125}$I-IAP (see Materials and methods). In four animals, distribution of $^{125}$I-IAP did not reach equilibrium within the time course of the experiment. In these cases, a value of 0.74 was assumed for $\lambda$. HPLC results showed that, after constant infusion of $^{125}$I-IAP for approximately an hour and a half, 89% of $^{125}$I tumour counts resided in the peak associated with IAP, 11% were associated with the alcohol-insoluble tissue fraction and <1% appeared in a lower molecular weight peak. These results indicate a small amount of binding and metabolism of the tracer in the tumour tissue. However, almost identical values were obtained for whole venous blood samples, such that $\lambda$ calculated from total $^{125}$I in tumour and blood was within 5% of the value calculated from $^{125}$I in the IAP fraction. The value of 0.74, calculated from total $^{125}$I counts in tumour and blood *in vivo*, was therefore considered to be a true measure of the tissue–blood partition coefficient for $^{125}$I-IAP in this tumour system. This compares with a value of 0.8 previously reported for $^{14}$C-IAP in the brain (Sakurada *et al*., 1978) and in subcutaneous rat tumours (Tozer & Morris, 1990).

Figure 4 shows the relationship between $VD_{app}$ and perfused fraction ($\alpha$) and the initial venous outflow for the whole group of 13 tumours. $VD_{app}$ tends to increase with increasing blood flow ($R = 0.70$, $P = 0.008$), suggesting that low blood flow is associated with poor access of $^{125}$I-IAP to all tumour regions (Figure 4a). However, despite a tendency for $\alpha$ to be lower at lower tumour blood flow, there was too much scatter in the data for this to be significant (Figure 4b). Figure 4b shows that $\alpha$ was within approximately 80% of the

**Figure 3** The early time courses of $^{125}$I-IAP uptake into the two tumours shown in Figure 2 (O) together with their respective arterial levels (×). These data represent $C_{tiss}(t)$ and $C_i(t)$ respectively in the calculation of $TBF_{IAP}$. The solid line represents the best fit through the tumour data. $TBF_{IAP}$ was calculated to be $0.435 \pm 0.012$ ml g$^{-1}$ min$^{-1}$ for tumour A and $0.244 \pm 0.009$ ml g$^{-1}$ min$^{-1}$ for tumour B. $VD_{app}$ was $0.743 \pm 0.005$ for tumour A and $0.578 \pm 0.009$ for tumour B.

**Figure 4** The relationship between $VD_{app}$ and venous outflow (**a**) and between perfused fraction α and venous outflow (**b**) for tissue-isolated preparations of the P22 tumour. Each point represents a single tumour. The solid line is the best straight-line fit through the data.

whole tumour volume for 10 out of 13 tumours. An unperfused fraction of 0–20% is consistent with tumour necrosis or possibly acutely ischaemic tumour regions (Chaplin *et al.*, 1987). An unperfused fraction of less than 50% was observed in a minority of cases (3 out of 13 tumours).

In a second group of 15 tumours, venous outflow was measured after removal of the tumour at the end of $TBF_{IAP}$ determination (measured over 30 s, see Materials and methods). Although there was a tendency for venous outflow to decrease with increase in tumour size, this was not significant for the number of tumours and the size range used (results not shown). Figure 5 shows that there was a significant residual venous outflow after the tumour had been removed. This flow must be either arteriovenous anastomoses or nutritive blood flow within the vascular pedicle which usually develops a fatty sheath during tumour growth. Mean residual flow for 13 animals shown in Figure 5 was $12.5 \pm 1.5\%$ of the total venous outflow measured immediately before removal of the tumour. Residual flow was not measureable in 2 out of 15 animals, and a value of 12.5% was used for these animals (not shown in Figure 5). The tendency for the percentage residual blood flow to decrease with increase in tumour size could be explained by a constant flow to the pedicle concomitant with an increasing total blood flow as tumour burden increases. However, this does not reach statistical significance with the numbers of animals used ($P = 0.098$).

Figure 6 shows the relationship between venous outflow,

**Figure 5** The relationship between residual blood flow in the vascular pedicle supplying the tumour and tumour weight. Each point represents a single tumour. The solid line is the best straight-line fit through the data.

**Figure 6** The relationship between tumour blood flow as measured by uptake of IAP ($TBF_{IAP}$) and tumour blood flow as measured by venous outflow from tissue-isolated tumours. Each point represents a single tumour. The solid line is the best straight-line fit through the data. The dashed line is the line of equivalence.

corrected for residual flow in the vascular pedicle, versus $TBF_{IAP}$. A linear relationship ($R = 0.84$) is significant ($P = 0.0004$). On average, $TBF_{IAP}$ was $94.8 \pm 4.4\%$ of the venous outflow. Venous outflow measurements will include the nutritive blood flow to the tumour as well as blood which is 'shunted' from the arterial to the venous side of the circulation with minimal exchange of solutes between blood and tumour tissue. $TBF_{IAP}$ will include very little of the shunted flow since the method relies on uptake of the radiotracer into the tissue. The small discrepancy between the two measurements therefore suggests that only a small percentage (approximately 5%) of blood passing through the tumour was shunted blood.

### Discussion

The tissue-isolated tumour preparation combined with radiotracer techniques provided a means for quantifying some of the characteristics of tumour blood flow. In particular, we were able to determine the total venous outflow from the tumour, the perfused fraction of the tumour and the amount of shunted blood which passes through the tumour without any significant nutrient exchange. Total venous outflow included a significant amount of blood flow to the vascular pedicle (approximately 10–20%) which can be minimised by using tumours as large as possible.

The perfused fraction of the tumour (α) was obtained from a comparison of $VD_{app}$ (estimated from data collected over a 400 s continuous infusion of IAP) and the corresponding equilibrium partition coefficient (λ). α was generally 80–100% of the tumour volume. This can be explained by necrosis and/or the presence of acutely ischaemic tumour regions, where low or non-existent perfusion would preclude local delivery of IAP to the tumour tissue. Our data were insufficient to determine whether increased venous outflow affected the perfused fraction. However, a separate study has shown that increasing the venous outflow above normal, by increasing the blood volume and therefore the perfusion pressure, has no effect on tumour vascular resistance, suggesting that this is not the case (unpublished data). It should also be noted that, in general, heterogeneity of local blood flow rates may lead to a low $VD_{app}$, as estimated using the single-compartment Kety model (Iida *et al.*, 1989). Heterogeneous blood flow is a characteristic feature of tumours and has been demonstrated in the P22 tumour using quantitative autoradiography (Tozer & Shaffi, 1993).

The shunted fraction was rather variable between individual tumours but, on average, only accounted for about 5% of the total venous outflow. This value is smaller than suggested in some previous publications which have relied on measurement of the fraction of arterially administered microspheres (average diameter approximately 15 μm) which escape trapping in the tumour microcirculation (Wheeler *et al.*, 1986; Tveit *et al.*, 1987). This method assumes that large tumour vessels play no part in nutrient exchange and thus may overestimate the true shunted fraction of blood. Wheeler *et al.* (1986), in their study of human head and neck cancer, also suggested that some of the shunting they observed (on average 23% of the total blood flow) may have been in normal tissue rather than in the tumour itself. Goldberg *et al.* (1991) have discussed the problems associated with using microspheres for measurement of shunted fraction and concluded that once errors such as leaching of radioactivity from the spheres and heterogeneity in sphere size were minimised the shunted fraction within human liver metastases could be reduced to less than 6%. Gullino and Grantham (1961*b*), who developed the original method for growing 'tissue-isolated' tumours in the ovarian site, used uptake of $^{42}KCl$ or $^{86}RbCl$ and Sapirstein's principles (Sapirstein, 1958) to determine blood flow to rat tumours growing in the ovary for comparison with the total venous outlfow. Their calculations included assumptions for the cardiac output of the host rats and they did not compare their tracer uptake method for blood flow with venous outflow in the same animals. However, in separate groups of animals, the mean blood flow values for the two methods were very similar, suggesting that there was minimal shunting of blood in these tumours. It is possible that tissue-isolated tumours contain less shunted blood than other tumours but this is a very difficult issue to address experimentally. For the P22 tumour, spatial heterogeneity of $TBF_{IAP}$ in tissue-isolated preparations is very similar to that in subcutaneous ones (unpublished data), suggesting that the vessel arrangement is similar for the two types. However, we cannot rule out the possibility that the tissue-isolated preparation *per se* is a major determinant of the degree of shunting present. Similarly, the influence of anaesthesia on the shunted fraction is unknown.

Taken together, evidence to data suggests that the amount of blood shunted through tumours, with minimal nutrient exchange, is less than previously supposed (Vaupel *et al.*, 1989). Since the amount of shunted blood in tumours has considerable implications for chemotherapy and the oxygenation of tumours, more studies are required to determine the degree of shunting in different tumour types.

For practical purposes, we have shown that measurement of total venous outflow is a good measurement of exchangeable tumour blood flow in our system, as long as a correction is made for the blood supply to the vascular pedicle. This method allows for on-line measurement of absolute blood flow in these tissue-isolated preparations. Tracer methods allow for only a single blood flow measurement but have the advantage that they can be adapted to provide microregional information by using quantitative autoradiography for assessment of tissue tracer levels (Tozer & Shaffi, 1993). Our blood flow calculations from tracer uptake kinetics include a correction for blood delayed and dispersed in the plastic cannulae used for collection of arterial blood. Failure to take these effects into account can cause significant errors (Iida *et al.*, 1986). The present study has shown that tumours commonly contain regions which are not readily accessible to diffusible tracers, resulting in a low apparent volume of distribution for the tracer at early times after injection. The true volume of distribution (or tissue–blood partition coefficient, λ) can only be obtained by equilibrium studies. An accurate measurement of volume of distribution is required for the application of these methods to tumour blood flow measurement.

We would like to thank Mike Stratford and Madeleine Dennis for performing the HPLC analysis, David Hirst for useful discussion and CRC Gray Laboratory staff for care of the animals. This work was supported by the Cancer Research Campaign.

### References

CHAPLIN, D.J., OLIVE, P.L. & DURAND, R.E. (1987). Intermittent blood flow in a murine tumor: radiobiological effects. *Cancer Res.*, **47**, 597–601.

DEWHIRST, M.W., TSO, C.Y., OLIVER, R., GUSTAFSON, C.S., SECOMB, T.W. & GROSS, J.F. (1989). Morphological and haemodynamic comparison of tumor and healing normal tissue microvasculature. *Int. J. Radiat. Oncol. Biol. Phys.*, **17**, 91–99.

ENDRICH, B., HAMMERSEN, F., GOTZ, A. & MESSMER, K. (1982). Microcirculatory blood flow, capillary morphology, and local oxygen pressure of the hamster amelanotic melanoma. A-Mel-3. *J. Natl Cancer Inst.*, **68**, 475–485.

FALK, P. (1980). The vascular pattern of the spontaneous C3H mouse mammary carcinoma and its significance in radiation response and in hyperthermia. *Eur. J. Cancer*, **16**, 203–217.

FOLKMAN, J. (1985). Tumour angiogenesis. *Adv. Cancer Res.*, **43**, 175–203.

GOLDBERG, J.A., THOMSON, J.A.K., CURACH, G., ANDERSON, J.H., WILLMOTT, N., BESSENT, R.G., MCKILLOP, J.H. & MCARDLE, C.S. (1991). Arteriovenous shunting in patients with colorectal liver metastases. *Br. J. Cancer*, **63**, 466–468.

GRANTHAM, F.H., HILL, D.M. & GULLINO, P.M. (1973). Primary mammary tumours connected to the host by a single artery and vein. *J. Natl Cancer Inst.*, **50**, 1381-1383.

GULLINO, P.M. & GRANTHAM, F.H. (1961*a*). Studies on the exchange of fluids between host and tumor. I. A method for growing 'tissue-isolated' tumors in laboratory animals. *J. Natl Cancer Inst.*, **27**, 679-693.

GULLINO, P.M. & GRANTHAM, F.H. (1961*b*). Studies on the exchange of fluids between host and tumor. II. The blood flow of hepatomas and other tumors in rats and mice. *J. Natl Cancer Inst.*, **27**, 1465-1491.

IIDA, H., KANNO, I., MIURA, S., MURAKAMI, M., TAKAHASHI, K. & UEMURA, K. (1986). Error analysis of a quantitative cerebral blood flow measurement using $H_2{}^{15}O$ autoradiography and positron emission tomography, with respect to the dispersion of the input function. *J. Cereb. Blood Flow Metab.*, **6**, 536-545.

IIDA, H., KANNO, I., MIURA, S., MURAKAMI, M., TAKAHASHI, K. & UEMURA, K. (1989). A determination of the regional brain/blood partition coefficient of water using dynamic positron emission tomography. *J. Cereb. Blood Flow Metab.*, **9**, 874-885.

KETY, S.S. (1960). Theory of blood tissue exchange and its application to measurements of blood flow. *Methods Med. Res.*, **8**, 223-227.

LAMMERTSMA, A.A., CUNNINGHAM, V.J., DEIBER, M.P., HEATHER, J.D., BLOOFIELD, P., NUTT, J., FRACKOWIAK, R.S.J. & JONES, T. (1990). Combination of dynamic and integral methods for generating reproducible functional CBF images. *J. Cereb. Blood Flow Metab.*, **10**, 675-686.

LAMMERTSMA, A.A., MARTIN, A.J., FRISTON, K.J. & JONES, T. (1992). *In vivo* measurement of the volume of distribution of water in cerebral grey matter: effects on the calculation of regional cerebral blood flow. *J. Cereb. Blood Flow Metab.*, **12**, 291-295.

NELDER, J.A. & MEAD, R. (1965). A simplex method for function minimisation. *Comp. J.*, **7**, 308-313.

RUBIN, R. & CASARETT, G.W. (1966). Microcirculation of tumours. I. Anatomy, function and necrosis. *Clin. Radiol.*, **17**, 220-229.

SAKURADA, O., KENNEDY, C., LEHLE, J., BROWN, J.D., CARBIN, J.L. & SOKOLOFF, L. (1978). Measurement of local cerebral blood flow with iodo [$^{14}C$] antipyrine. *Am. J. Physiol.*, **234**, H59-H66.

SENSKY, P.L., PRISE, V.E., TOZER, G.M., SHAFFI, K.M. & HIRST, D.G. (1993). Resistance to flow through tissue-isolated transplanted rat tumours located in two different sites. *Br. J. Cancer*, **67**, 1337-1341.

SAPIRSTEIN, L.A. (1958). Regional blood flow by fractional distribution of indicators. *Am. J. Physiol.*, **193**, 161-168.

SHUBIK, P. (1982). Vascularization of tumors: a review. *J. Cancer Res. Clin. Oncol.*, **103**, 211-226.

TOZER, G.M. & MORRIS, C. (1990). Blood flow and blood volume in a transplanted rat fibrosarcoma: comparison with various normal tissues. *Radiother. Oncol.*, **17**, 153-166.

TOZER, G.M. & SHAFFI, K.M. (1993). Modification of tumour blood flow using the hypertensive agent, angiotensin II. *Br. J. Cancer*, **67**, 981-988.

TOZER, G.M., LEWIS, S., MICHALOWSKI, A. & ABER, V. (1990). The relationship between regional variations in blood flow and histology in a transplanted rat fibrosarcoma. *Br. J. Cancer*, **61**, 250-257.

TVEIT, K., WEISS, L., LUNDSTAM, S. & HULTBORN, R. (1987). Perfusion characteristics and norepinephrine reactivity of human renal carcinoma. *Cancer Res.*, **47**, 4709-4713.

VAUPEL, P., KALLINOWSKI, F. & OKUNIEFF, P. (1989). Blood flow, oxygen and nutrient supply, and metabolic environment of human tumors: a review. *Cancer Res.*, **49**, 6449-6465.

WARREN, B.A. (1979). The vascular morphology of tumors. In *Tumor Blood Circulation: Angiogenesis, Vascular Morphology and Blood Flow of Experimental and Human Tumors*, Peterson, H.-I. (ed.) pp. 1-48. CRC Press: Boca Raton, FL.

WHEELER, R.H., ZIESSMAN, H.A., MEDVEC, B.R., JUNI, J.E., THRALL, J.H., KEYES, J.W., PITT, S.R. & BAKER, S.R. (1986). Tumor blood flow and systemic shunting in patients receiving intrarterial chemotherapy for head and neck cancer. *Cancer Res.*, **46**, 4200-4204.

WILKINSON, G.N. (1961). Statistical estimations in enzyme kinetics. *Biochem. J.*, **80**, 324-333.


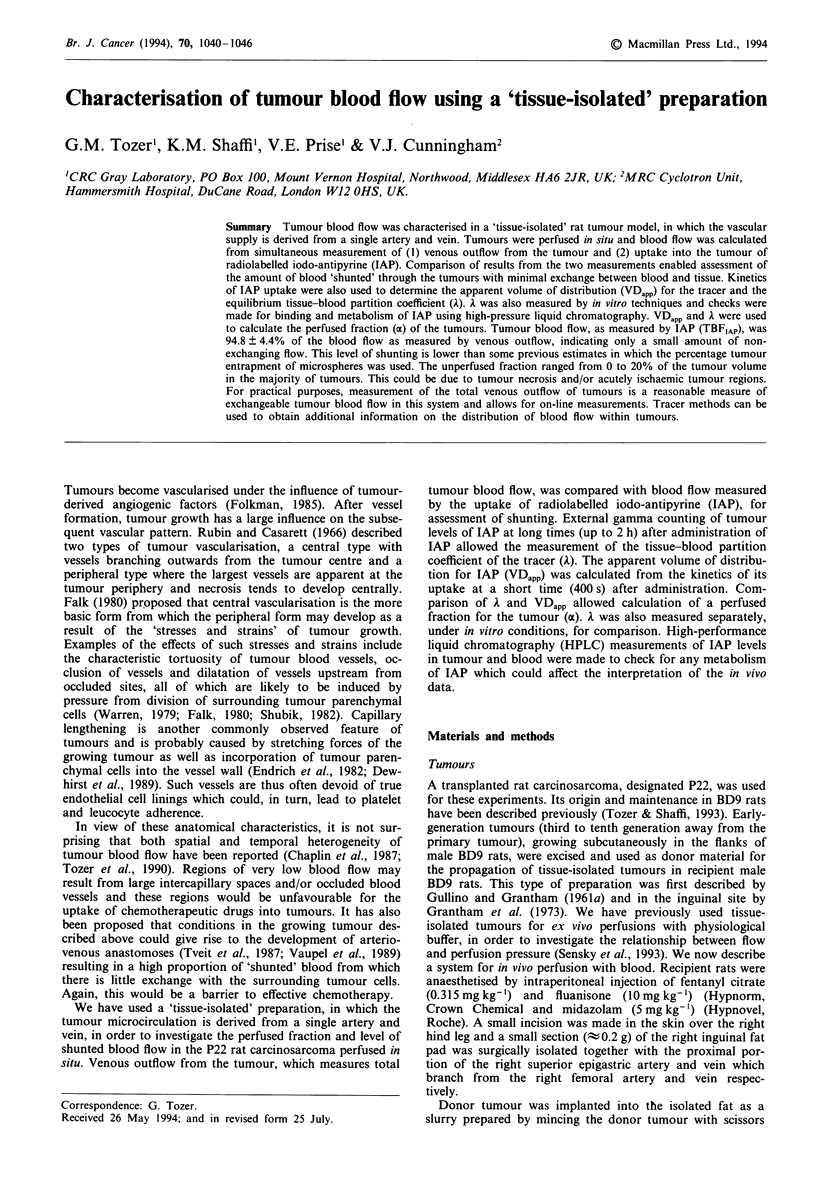

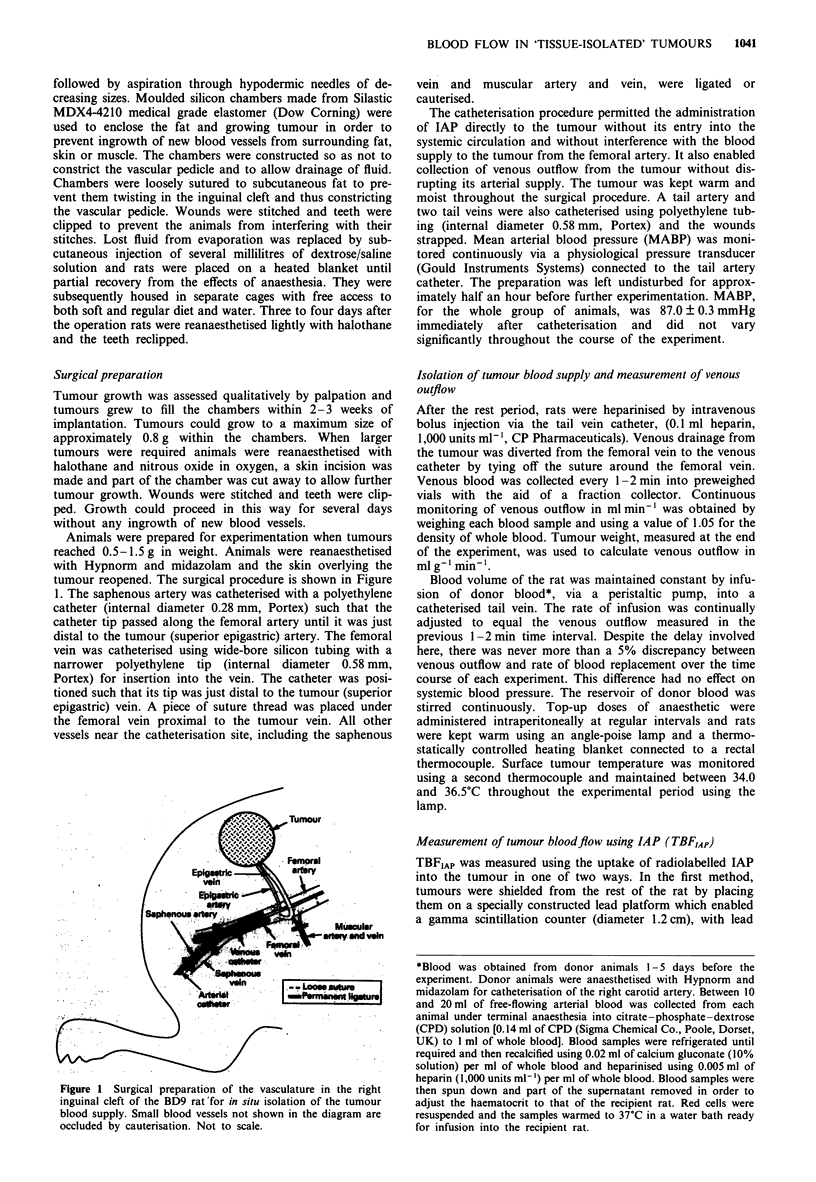

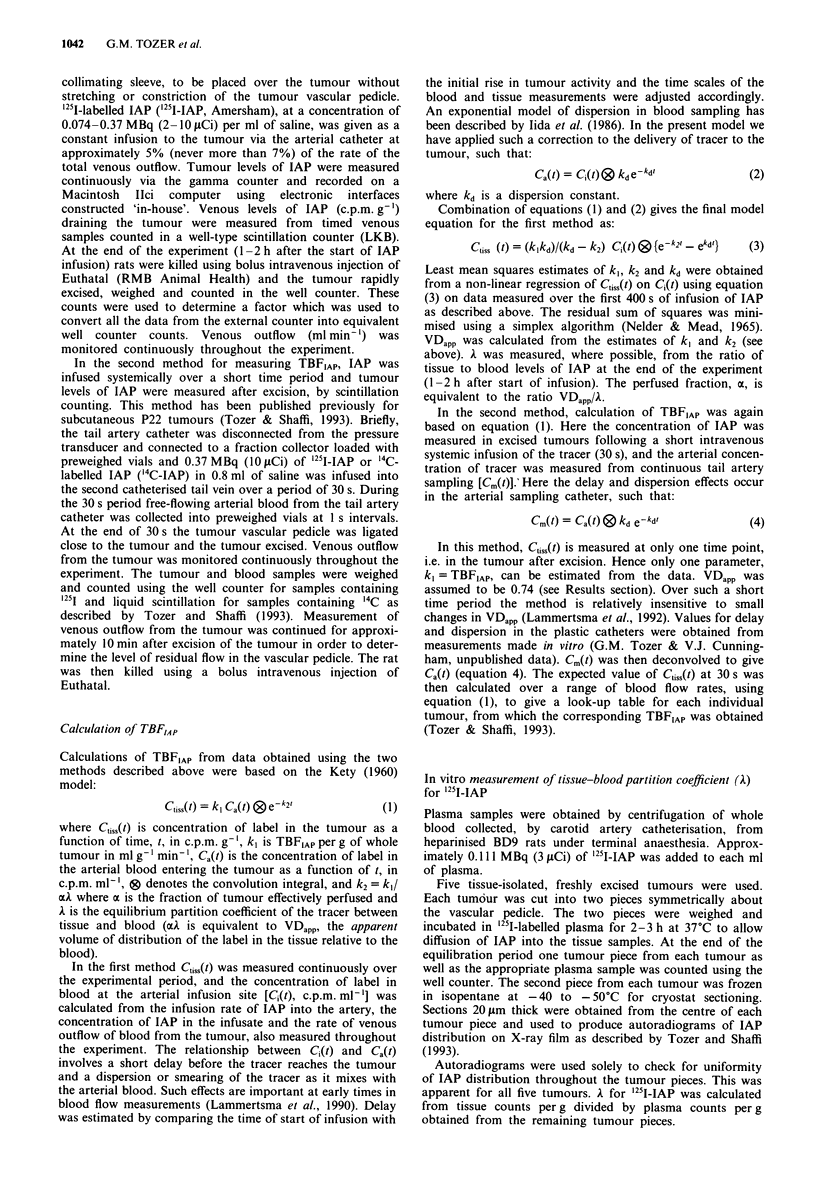

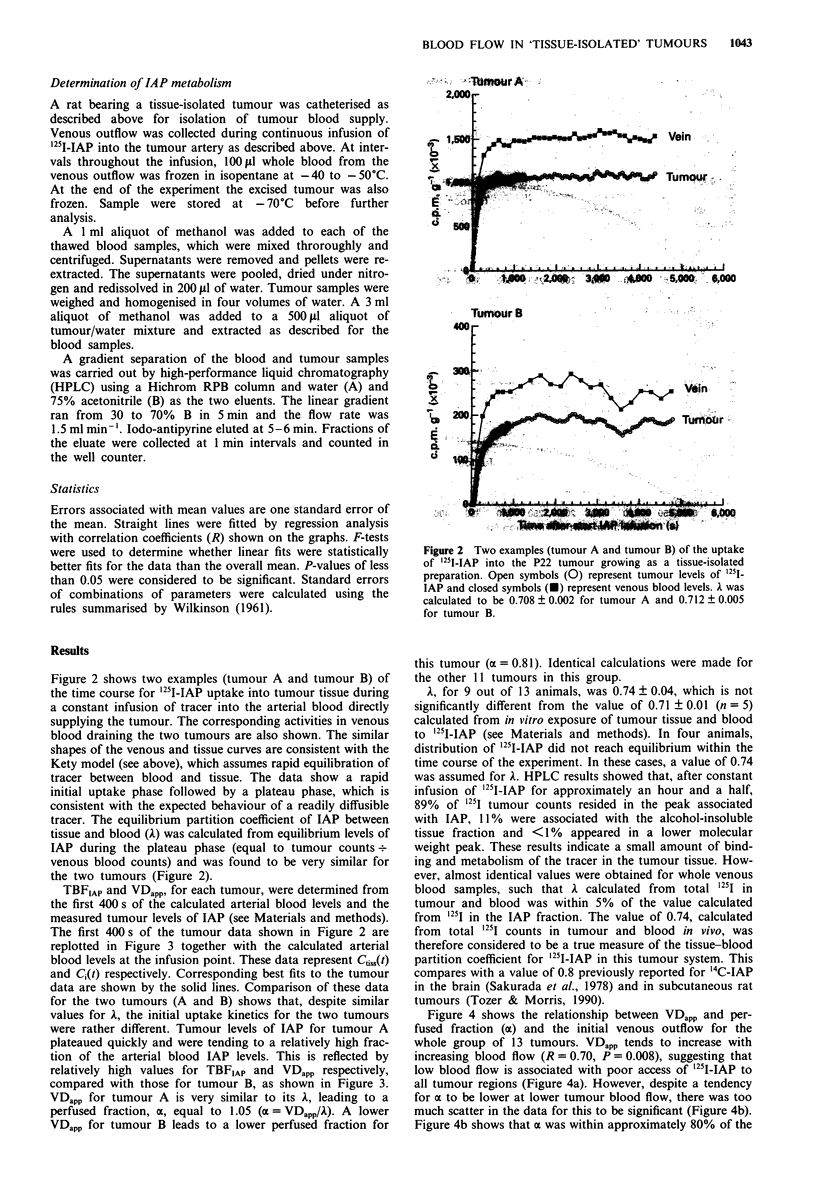

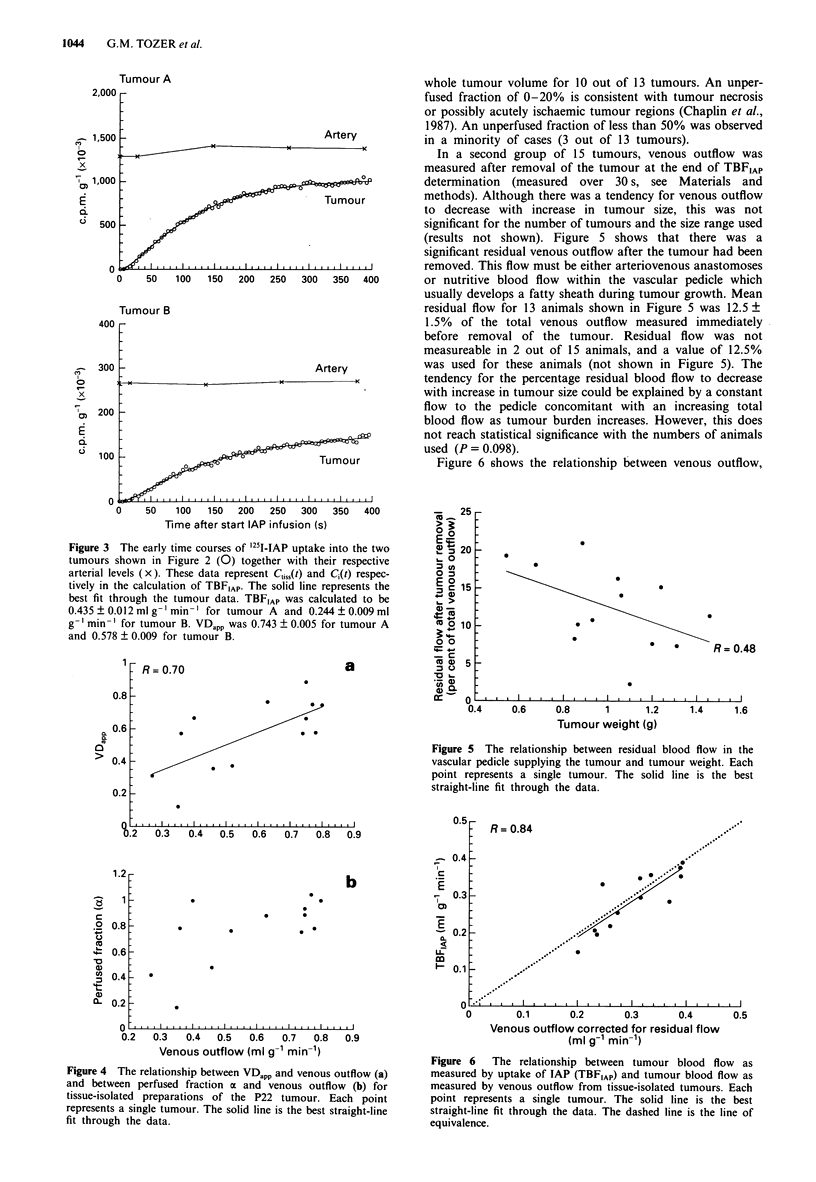

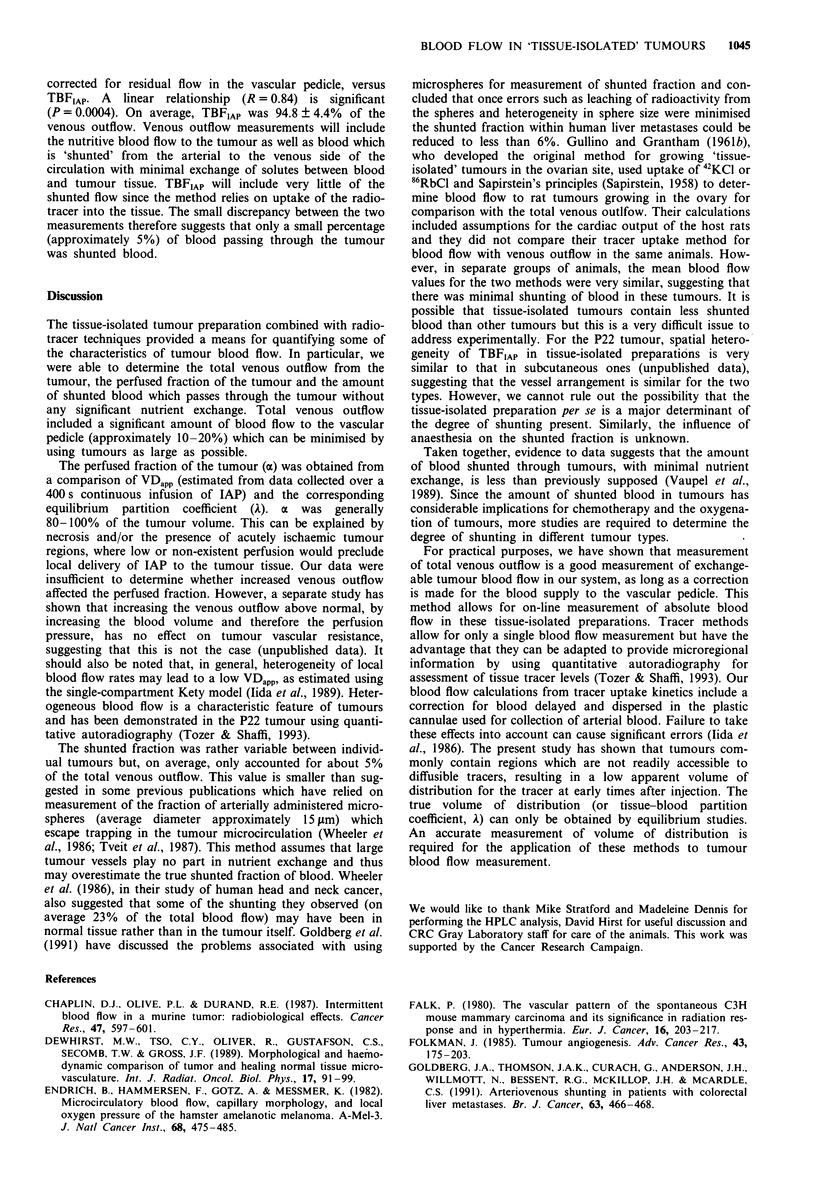

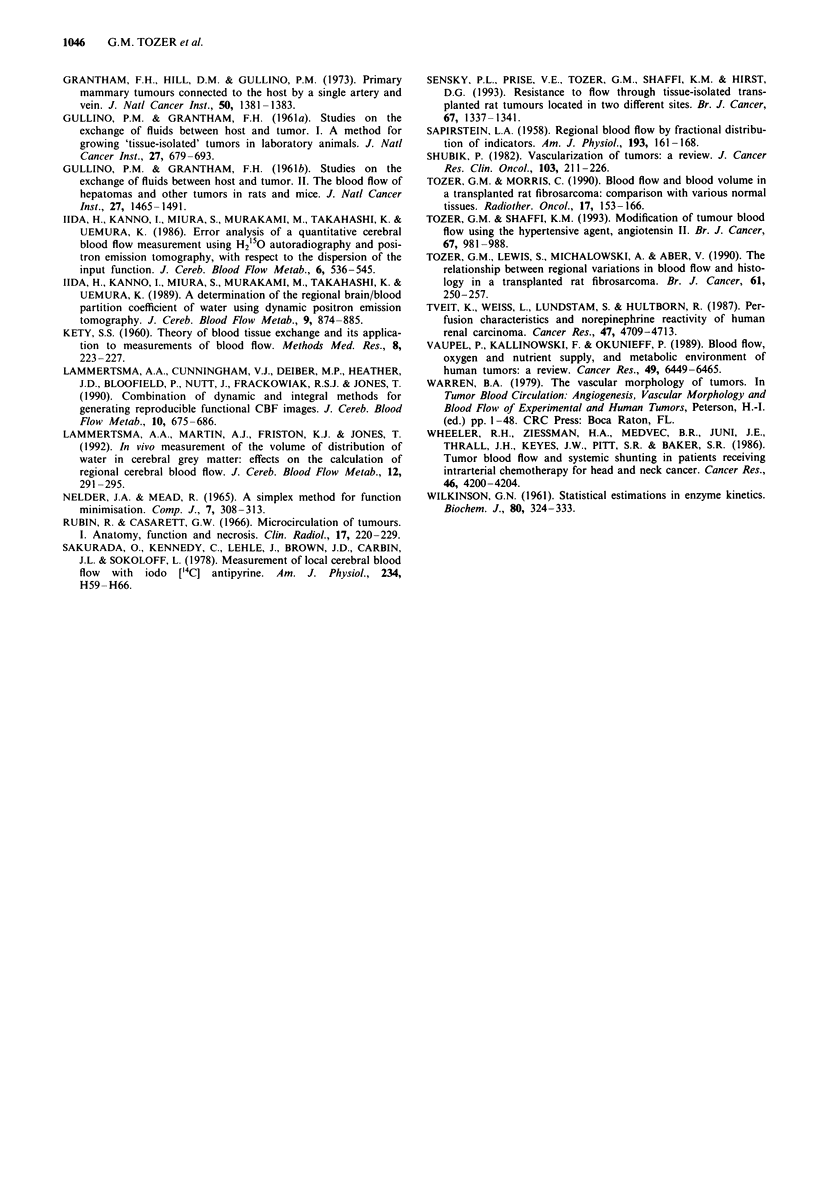


## References

[OCR_00983] Chaplin D. J., Olive P. L., Durand R. E. (1987). Intermittent blood flow in a murine tumor: radiobiological effects.. Cancer Res.

[OCR_00988] Dewhirst M. W., Tso C. Y., Oliver R., Gustafson C. S., Secomb T. W., Gross J. F. (1989). Morphologic and hemodynamic comparison of tumor and healing normal tissue microvasculature.. Int J Radiat Oncol Biol Phys.

[OCR_00994] Endrich B., Hammersen F., Götz A., Messmer K. (1982). Microcirculatory blood flow, capillary morphology and local oxygen pressure of the hamster amelanotic melanoma A-Mel-3.. J Natl Cancer Inst.

[OCR_01000] Falk P. (1980). The vascular pattern of the spontaneous C3H mouse mammary carcinoma and its significance in radiation response and in hyperthermia.. Eur J Cancer.

[OCR_01005] Folkman J. (1985). Tumor angiogenesis.. Adv Cancer Res.

[OCR_01022] GULLINO P. M., GRANTHAM F. H. (1961). Studies on the exchange of fluids between host and tumor. I. A method for growing "tissue-isolated" tumors in laboratory animals.. J Natl Cancer Inst.

[OCR_01028] GULLINO P. M., GRANTHAM F. H. (1961). Studies on the exchange of fluids between host and tumor. II. The blood flow of hepatomas and other tumors in rats and mice.. J Natl Cancer Inst.

[OCR_01009] Goldberg J. A., Thomson J. A., McCurrach G., Anderson J. H., Willmott N., Bessent R. G., McKillop J. H., McArdle C. S. (1991). Arteriovenous shunting in patients with colorectal liver metastases.. Br J Cancer.

[OCR_01017] Grantham F. H., Hill D. M., Gullino P. M. (1973). Primary mammary tumors connected to the host by a single artery and vein.. J Natl Cancer Inst.

[OCR_01041] Iida H., Kanno I., Miura S., Murakami M., Takahashi K., Uemura K. (1989). A determination of the regional brain/blood partition coefficient of water using dynamic positron emission tomography.. J Cereb Blood Flow Metab.

[OCR_01034] Iida H., Kanno I., Miura S., Murakami M., Takahashi K., Uemura K. (1986). Error analysis of a quantitative cerebral blood flow measurement using H2(15)O autoradiography and positron emission tomography, with respect to the dispersion of the input function.. J Cereb Blood Flow Metab.

[OCR_01052] Lammertsma A. A., Cunningham V. J., Deiber M. P., Heather J. D., Bloomfield P. M., Nutt J., Frackowiak R. S., Jones T. (1990). Combination of dynamic and integral methods for generating reproducible functional CBF images.. J Cereb Blood Flow Metab.

[OCR_01059] Lammertsma A. A., Martin A. J., Friston K. J., Jones T. (1992). In vivo measurement of the volume of distribution of water in cerebral grey matter: effects on the calculation of regional cerebral blood flow.. J Cereb Blood Flow Metab.

[OCR_01070] Rubin P., Casarett G. (1966). Microcirculation of tumors. I. Anatomy, function, and necrosis.. Clin Radiol.

[OCR_01085] SAPIRSTEIN L. A. (1958). Regional blood flow by fractional distribution of indicators.. Am J Physiol.

[OCR_01073] Sakurada O., Kennedy C., Jehle J., Brown J. D., Carbin G. L., Sokoloff L. (1978). Measurement of local cerebral blood flow with iodo [14C] antipyrine.. Am J Physiol.

[OCR_01079] Sensky P. L., Prise V. E., Tozer G. M., Shaffi K. M., Hirst D. G. (1993). Resistance to flow through tissue-isolated transplanted rat tumours located in two different sites.. Br J Cancer.

[OCR_01089] Shubik P. (1982). Vascularization of tumors: a review.. J Cancer Res Clin Oncol.

[OCR_01103] Tozer G. M., Lewis S., Michalowski A., Aber V. (1990). The relationship between regional variations in blood flow and histology in a transplanted rat fibrosarcoma.. Br J Cancer.

[OCR_01093] Tozer G. M., Morris C. C. (1990). Blood flow and blood volume in a transplanted rat fibrosarcoma: comparison with various normal tissues.. Radiother Oncol.

[OCR_01098] Tozer G. M., Shaffi K. M. (1993). Modification of tumour blood flow using the hypertensive agent, angiotensin II.. Br J Cancer.

[OCR_01109] Tveit E., Weiss L., Lundstam S., Hultborn R. (1987). Perfusion characteristics and norepinephrine reactivity of human renal carcinoma.. Cancer Res.

[OCR_01114] Vaupel P., Kallinowski F., Okunieff P. (1989). Blood flow, oxygen and nutrient supply, and metabolic microenvironment of human tumors: a review.. Cancer Res.

[OCR_01132] WILKINSON G. N. (1961). Statistical estimations in enzyme kinetics.. Biochem J.

[OCR_01125] Wheeler R. H., Ziessman H. A., Medvec B. R., Juni J. E., Thrall J. H., Keyes J. W., Pitt S. R., Baker S. R. (1986). Tumor blood flow and systemic shunting in patients receiving intraarterial chemotherapy for head and neck cancer.. Cancer Res.

